# Sustained clinical success at 7-year follow-up after arthroscopic Lift-Drill-Fill-Fix (LDFF) of primary osteochondral lesions of the talus

**DOI:** 10.1007/s00167-022-07243-5

**Published:** 2023-01-05

**Authors:** Quinten G. H. Rikken, J. Nienke Altink, Jari Dahmen, Kaj T. A. Lambers, Sjoerd A. S. Stufkens, Gino M. M. J. Kerkhoffs

**Affiliations:** 1 Department of Orthopedic Surgery and Sports Medicine, Amsterdam UMC, University of Amsterdam, Meibergdreef 9, Amsterdam, The Netherlands; 2 Amsterdam Movement Sciences, Musculoskeletal Health, Amsterdam, The Netherlands; 3 Academic Center for Evidence Based Sports Medicine (ACES), Amsterdam UMC, Amsterdam, The Netherlands; 4 Amsterdam Collaboration for Health and Safety in Sports (ACHSS), International Olympic Committee (IOC) Research Center, Amsterdam UMC, Amsterdam, The Netherlands; 5 Department of Orthopedic Surgery, Amphia Hospital, Breda, the Netherlands

**Keywords:** Osteochondral lesion, OLT, Fixation, LDFF, Surgery

## Abstract

**Purpose:**

To describe the long-term clinical results of arthroscopic fragment fixation for chronic primary osteochondral lesions of the talus (OLT), using the Lift-Drill-Fill-Fix (LDFF) technique.

**Methods:**

Eighteen patients (20 ankles) underwent fixation for a primary OLT with an osteochondral fragment using arthroscopic LDFF and were evaluated at a minimum of 5-year follow-up. Pre- and postoperative clinical assessment was prospectively performed by measuring the Numeric Rating Scale (NRS) of pain at rest, during walking and when running. Additionally, the change in Foot and Ankle Outcome Score (FAOS) and the procedure survival (i.e., no reoperation for the OLT) at final follow-up was assessed.

**Results:**

At a mean follow-up of 7 years, the median NRS during walking significantly improved from 7 (IQR 5–8) pre-operatively to 0 (IQR 0–1.5) at final follow-up (*p* =  < 0.001). This result was sustained from 1-year follow-up to final follow-up. The NRS during running significantly improved from 8 (IQR 6−10) to 2 (IQR 0–4.5) (*p* < 0.001) and the NRS in rest from 2.5 (IQR 1–3) to 0 (IQR 0–0) (*p* =  < 0.001). The median FAOS at final follow-up was 94 out of 100 for pain, 71 for other symptoms, 99 for activities of daily living, 80 for sport and 56 for quality of life. The FOAS remained significantly improved post-operatively on all subscales, except for the symptoms subscale. The procedure survival rate is 87% at final follow-up.

**Conclusion:**

Arthroscopic LDFF for fixable chronic primary OLTs results in excellent pain reduction and improved patient-reported outcomes, with sustained results at long-term follow-up. These results indicate that surgeons may consider arthroscopic LDFF as treatment of choice for fragmentous OLT.

**Level of evidence:**

Level IV, prospective case series.

## Introduction

Osteochondral lesions of the talus (OLT) affect the articular cartilage and its underlying subchondral bone. Treatment of these lesions is challenging as no superior treatment is available to date [2]. Treatment of OLTs should be based on lesion and patient characteristics in a patient individualized, evidence-based, shared decision-making process [20]. Choosing between surgical options is mainly directed by lesion morphology [19]. Patients presenting with an osteochondral fragmentous lesion may benefit from fragment fixation both in the acute (< 6 weeks after trauma) or chronic phase.

The theoretical advantage of fixation over other surgical techniques is the preservation of native hyaline cartilage, immediate stabilization of the fragment and restoration of the talar dome congruency, as well as facilitating subchondral bone healing [8, 18]. One such a fixation technique for OLTs is “Lift-Drill-Fill-Fix” (LDFF) [8]. The LDFF technique is indicated for primary fragmentous OLTs with ≥ 10 mm in diameter and ≥ 3 mm thickness [8, 18]. In the case of a chronic OLT, the LDFF procedure can be considered an intra-articular non-union repair by means of subchondral bone drilling, autologous bone grafting, and compression. LDFF can be performed both open and arthroscopically, with previous studies reporting excellent short- to mid-term clinical outcomes [8, 10]. In general, mid-term outcomes of fragment fixation for OLTs are promising [6, 9, 10, 17, 23]. The durability and longevity of such fixation procedures remain a matter of debate however [3, 9].

The primary aim of the present study is therefore to evaluate the long-term patient-reported outcomes of arthroscopic LDFF. The hypothesis is that the results of arthroscopic LDFF results are maintained over time. The secondary aims of this study are to investigate the survival rate and complications.

## Materials and methods

Approval for this study was obtained from the Medical Ethical Committee of Amsterdam UMC, Location AMC (reference number: MEC 08/326). The present study is in accordance with the Medical Research Involving Human Subjects Act (WMO) and the principles of the Declaration of Helsinki.

### Patient selection

The present study is a long-term follow-up of a cohort of consecutive patients who underwent arthroscopic fixation by means of LDFF for a primary chronic (> 6 weeks after trauma or start of symptoms) OLT and minimum of 6-month conservative treatment before surgery [10, 20]. Fragmentous OLTs, with a preferred minimum diameter of 10 mm (mm), 3 mm of fragment thickness, and reachable by arthroscopy were considered fixable [10, 19]. The exclusion criteria as well as the surgical technique and postoperative rehabilitation protocol for arthroscopic LDFF were described by Lambers et al. [10]. Additionally, patients who were lost to follow-up, patients who declined to participate, patients who underwent surgery of the lower extremity within 6 months before final follow-up, and patients with less than 5-year follow-up were excluded in the present study. Patients were identified and included in a cross-sectional manner after applying the inclusion and exclusion criteria.

### Outcome measures

Outcomes were collected prospectively at the preoperative, one- and two-year follow-ups, and at a minimum of 5 years postoperatively at the cross-sectional final follow-up [10]. At final follow-up, patients were contacted by phone to obtain informed consent for the present study.

#### Clinical outcomes

To obtain patient-reported outcome measures (PROMs), online questionnaires were distributed via the Castor^©^ electronic data capture system. The primary outcome measure for the present study was the Numeric Rating Scale (NRS) of pain during walking, which is a patient-reported pain scale from 0 (no pain) to 10 (most pain imaginable) [5]. The NRS contained two additional subscales in the present study, namely the NRS in rest, and the NRS during running. Secondary patient-reported outcome measures collected were the Foot and Ankle Outcome Score (FAOS) and the Short Form Health Survey (SF-36) [1, 25].

By phone, and from the electronic patient records, any reoperation of the ankle was recorded. Revision surgery was defined as any reoperation of the OLT after the arthroscopic LDFF procedure. The survival rate was defined as the proportion of the number of ankles which did not undergo revision surgery from the total number of ankles included at final follow-up. Postoperative complications were extracted from the hospital electronic patient records.

#### Radiological outcomes

All patients received a preoperative and one-year postoperative radiological assessment of the OLT by means of a computed tomography (CT) scan. The aforementioned study by Lambers et al. [10] previously assessed the osteochondral fragment union rate and the subchondral bone plate appearance at one-year follow-up, these outcomes were therefore not included in the present study. Radiological lesion baseline characteristics included: lesion size (as measured in millimeters from three planes—anterior–posterior (AP), medial–lateral (ML) directions, and depth), the number of lesions per ankle, the presence of cysts, and the lesion location according to Raikin et al. [16]. CT measurements were performed by two independent measurers (Q.R. and J.N.A.), in case of disagreement, a third assessor (*J.D.) was decisive. Lesion size is reported as the mean lesion size from the two independent assessors.

### Data collection

Baseline patient and treatment characteristics were extracted from the hospital electronic patient records. Patient characteristics included sex, laterality, age at surgery, body mass index (BMI), participation in sports and level of sports participation (i.e., none, amateur, competitive, or professional), injury circumstances (i.e., traumatic-fracture or sprain- or non-traumatic onset), and concomitant injuries. Treatment characteristics collected were the follow-up time, screw type used for fixation (i.e., bio-absorbable screw, chondral dart, or cortical screw), and any concomitant procedures at index surgery.

### Statistical analysis

Before the start of the present study, a sample size calculation for the primary outcome was performed with a Wilcoxon signed-rank test, using a level of significance (*α*) of 0.05 (nQuery advisor 7.0, Statistical Solutions Ltd., Boston, MA). A minimally clinical important difference (MCID) of the NRS pain during walking 2.0 points between the preoperative and postoperative situation, with a standard deviation of 2.5 points and a power of 80% was chosen, as previous studies observed a much better improvement in pain [4, 10, 14, 22]. A minimum of 16 patients were needed.

Descriptive analysis was performed to summarize the baseline characteristic variables, which are presented with frequencies and percentages for categorical variables and means with standard deviations and ranges for continuous variables. Data were assessed visually for normality and with a Shapiro–Wilk test. Wilcoxon signed-rank test was used to compare clinical outcomes pre-operatively with postoperatively. Specifically for the primary outcome, a comparison of the NRS during walking between the final follow-up with 6-month follow-up, 1-year follow-up, and 2-year follow-up was made using the Wilcoxon signed-rank test. To test inter-rater reliability of the lesion size measurements and the lesion localization, 2-way mixed effects interclass correlation coefficient (ICC) model with absolute agreement and Cohen’s Kappa analysis were used, respectively. ICC analysis outcomes were interpreted according to Shrout et al. [24] with 0.41–0.60 being a fair agreement, 0.61–0.80 moderate agreement, and 0.81–1.00 substantial agreement. The agreement on the Cohen’s kappa test was interpreted as substantial if *k* = 0.61–0.8, and almost perfect if *k* > 0.81 [11]. A two-sided level of *P* < 0.05 was considered significant. All data analyses were conducted using Stata 15 (StataCorp LP, College Station, TX).

## Results

**Fig. 1:**
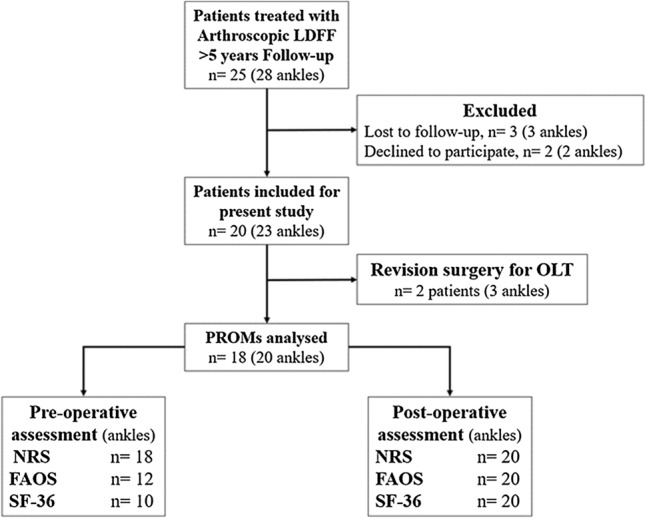
Flowchart of patient selection according to inclusion and exclusion criteria, *Of note*: pre-operative clinical outcome measures were not available for all patients

From the 25 eligible patients, 20 patients were included with a total of 23 ankles. 5 patients were excluded, as outlined in Fig. 1. Two patients underwent revision surgery for a total of 3 ankles (one bilateral case) and the PROMs were therefore analyzed for a total of 18 patients with 20 ankles at final follow-up. The baseline patient and lesion characteristics are available in Table 1. In terms of prior or concomitant surgical procedures to the ankle, one patient underwent an anterior arthroscopy for debridement of anteromedial soft-tissue impingement and a chondral lesion of the medial tibial plafond 12 months prior to the LDFF procedure and one patient received an additional Duquennoy procedure at the time of LDFF due to lateral ankle instability.

**Table 1:** Baseline patient- and lesion characteristics*

Patient characteristics	Total *N* = 18
Sex, *n* (% male)	9 (50%)
Age (years), mean ± SD (range)	24.2 ± 15.2 (11.3–62.2)
FU (months), mean ± SD (range)	82.9 ± 9.3 (71.4–96.4)
BMI (kg/m^2^), mean ± SD (range)	22.9 ± 3.7 (19.4–36.5)
History of smoking, *n* (%)	3 (17%)
Laterality, n (%) Right/Left/Bilateral	9 (50%) / 7 (39%) / 2 (11%)
Previous ankle trauma, *n* (%)	10 (56%)
Previous ankle fracture, *n* (%)	1 (6%)
Sports participation, *n* (%) Yes/No/Unknown	14 (78%) / 3 (17%) / 1 (5%)
Sports Level, *n* (%)	
- Professional	0 (0%)
- Competitive	9 (64%)
- Recreational	4 (29%)
- Unknown	1 (7%)
Concomitant procedures, *n* (%)	
- Open lateral ligament repair	1 (5%)

Lesion characteristics	Total *N* = 20
Presence of Cyst, *n* (%)	6 (30%)
Size (mm), mean ± SD (range)	
Anterior–Posterior	13.8 ± 2.9 (10.0–20.0)
Medial–Lateral	9.4 ± 2.5 (5.2–14.0)
Depth	7.0 ± 2.2 (4.0–11.5)
Location per zone^†^, *n* (%)	
Anteromedial (zone 1)	1 (5%)
Anterocentral (zone 2)	0
Anterolateral (zone 3)	2 (10%)
Centeromedial (zone 4)	15 (70%)
Central (zone 5)	0
Centerolateral (zone 6)	1 (5%)
Posteromedial (zone 7)	2 (10%)
Posterocentral (zone 8)	0
Posterolateral (zone 9)	0

Please note that the patient characteristics are given for the total number of patients (*n* = 18) who underwent post-operative clinical assessment (i.e., no failure casus), and that lesion characteristics are given for the total number of ankles (*n* = 20)

*n* number of, *SD* standard deviation, *FU* follow-up, *BMI* body mass index, mm millimeters

^†^One ankle had two lesions and was thus counted double

### Clinical outcomes

**Fig. 2:**
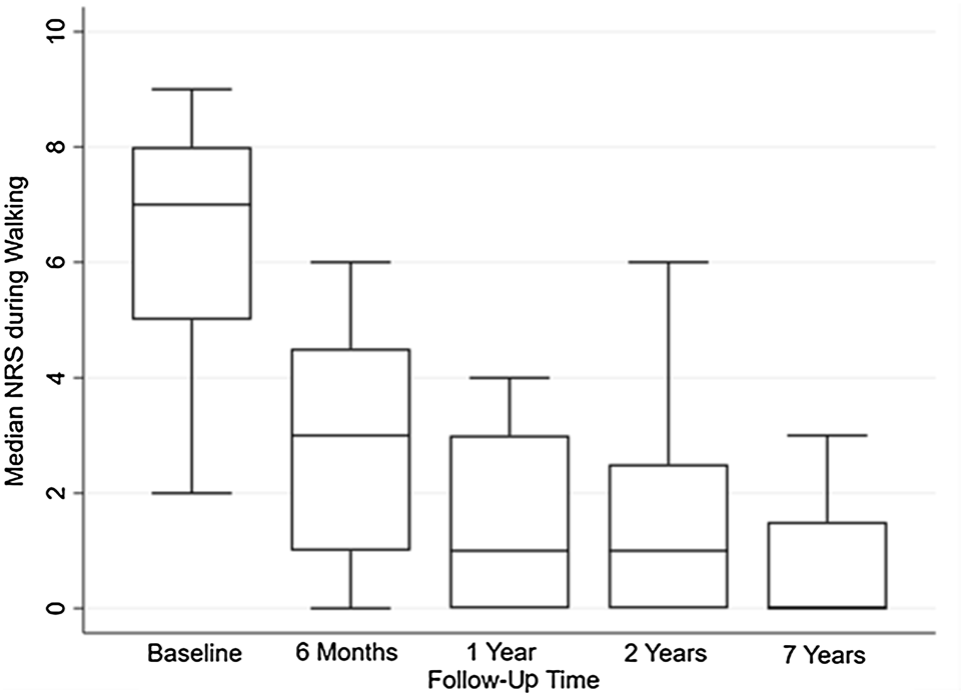
Boxplots of median NRS pain during walking over time, *Of note*: the following number of ankles available for analysis at certain time points; baseline *n* = 18, 6 months *n* = 12, 1 year *n* = 15, 2 years *n* = 20, 7 years *n* = 20

The primary outcome, the NRS pain during walking, significantly improved at mean 6.9-year follow-up (median: 0 out of 10 (IQR: 0–1.5)) compared to pre-operatively (median: 7 out of 10 (IQR: 5–8), *P* =  < 0.01), as well as for the other NRS subdomains (Table 2). The NRS during walking did not significantly change from 1-year postoperatively compared to the 2-year and long-term follow-up time points (Fig. 2). An overview of the PROMs is available in Table 2.

**Table 2:** Preoperative and final follow-up patient-reported clinical outcome scores

Outcome	Preoperative *n* = 18	Final follow-up *n* = 2*0*	*P* value
NRS, median (IQR)			
Pain (rest)	2.5 (1.0–3.0)	0.0 (0.0–0.0)	** < 0.01**
Pain (walking)	7.0 (5.0–8.0)	0.0 (0.0–1.5)	** < 0.01**
Pain (running)	8.0 (6.0–10.0) *n* = 13	2.0 (0.0–4.5)	** < 0.01**
FAOS, median (IQR) *n* = 12			
Symptoms	69.5 (52.0–75.0)	71.4 (57.1–84.0)	n.s
Pain	66.5 (54.0–79.5)	94.4 (83.3–100)	** < 0.01**
ADL	90.5 (74.5–97.0)	98.5 (97.1–100)	**0.02**
Sport	40.0 (27.5–60.0)	80.0 (60.0–100)	**0.01**
QoL	22.0 (13.0–34.5)	56.3 (50.0–68.8)	**0.02**
SF-36, median (IQR) *n* = 10			
PCS	43.3 (35.8–51.1)	45.1 (41.8–47.8)	n.s
MCS	57.1 (53.5–60.8)	37.4 (35.4–39.5)	** < 0.01**

*n* number of ankles, *NRS* Numeric Rating Scale, *IQR* Inter Quartile Range, *ADL* Activities of Daily Living, *QoL* Quality of Life, *SF-36* = Short Form-36, *PCS* Physical Component Summary, *MCS* Mental Component Summary, *n.s.* non-significant

### Reoperations, revision surgery, and complications

A total of two patients underwent surgery to the ankle unrelated to the OLT after LDFF. From these two patients, one had an anterior ankle arthroscopy for anteromedial soft-tissue impingement debridement and one had a gastrocnemius release at another institution at 18 and 86 months after the LDFF procedure, respectively. Additionally, two patients underwent surgery of the ipsilateral lower extremity not involving the ankle (one anterior cruciate ligament reconstruction and one bi-planar chevron osteotomy for a symptomatic hallux valgus).

**Fig. 3:**
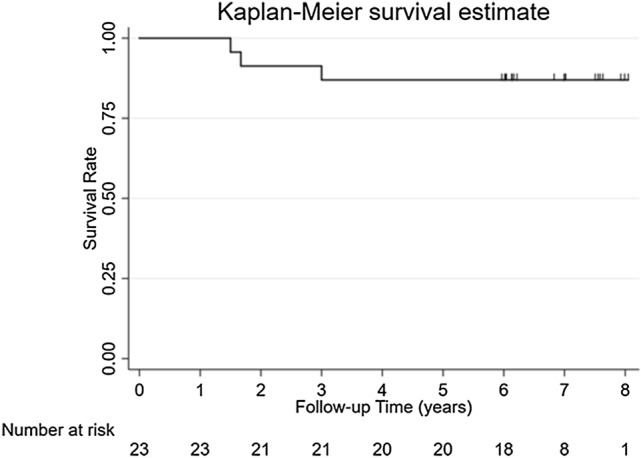
Kaplan–Meier survival curve for procedure survival over time, *Of note*: patients were censored starting at 5 year follow-up due to no long follow-up

From the 20 patients with 23 ankles available in this study, two patients with a total of three ankles (13%) underwent revision surgery. This corresponds to a procedure survival rate of 87% (Fig. 3). The baseline demographics and treatment characteristics, including reason for revision surgery, of these patients are provided in the Appendix. No complications were recorded in this cohort.

### Radiological assessment

One-year postoperative CT scans were performed for all ankles. The baseline lesion characteristics are reported in Table 1. The inter-rater reliability was observed to be substantial for the AP (ICC: 0.88), ML (ICC: 0.91), and depth (ICC: 0.82) lesion size measurements. The Cohens’ kappa for the inter-rater reliability of the lesion location was 0.71 (substantial).

## Discussion

The principal finding of this study is that clinical outcomes remain excellent at long-term follow-up in patients who underwent fixation of a chronic primary fragmentous OLT by means of arthroscopic LDFF. Moreover, the procedure survival rate is 87% at mean 7-year follow-up. These results indicate that outcomes of arthroscopic LDFF stand the test of time and that fixation may be considered for primary OLTs which are amendable for fixation.

To the knowledge of the authors, this is the first study reporting on the long-term clinical outcomes of arthroscopic fixation for OLTs. When comparing the outcomes of the present study to the literature, it is clear that, although fixation outcomes are reported, long-term outcomes and results from arthroscopic fixation are rare [3, 6, 9, 10, 12, 15, 17]. Overall, patient-reported clinical outcomes after open fixation available in the literature can be considered excellent and fragment union is seen from 77% in up to 100% of cases [6, 9, 12, 15, 17, 23]. To date, two studies reported on the long-term outcomes of open fixation. Kumai et al. [9] reported good Berndt and Harty scores in 16 and fair 3 in patients with more than 5-year follow-up. Dunlap et al. [3] observed a mean postoperative AOFAS score of 86, and good Berndt and Harty scores in 4 out of 5 patients at a mean 12 years of follow-up.

The advantage of fixation over other surgical treatment options for fragmentous OLTs is the preservation of the native hyaline cartilage, immediate stabilization of the fragment and restoration of the talar dome congruency, as well as facilitating subchondral bone healing [8, 18]. The LDFF procedure complements this by subchondral drilling and autologous bone grafting, essentially qualifying it as an intra-articular non-union repair. The superior subchondral bone healing following LDFF is supported by findings from Reilingh et al. [18], who observed that the subchondral bone healing after fixation is superior compared to bone marrow stimulation (BMS), the most frequent surgical treatment for OLTs [2]. However, when interpreting the revision rate of 7% reported in the literature for BMS at long-term follow-up [21], one can argue this can be considered comparable to the survival rate reported in the present study. Data concerning surgical treatment for fragmentous OLTs are too scarce, however, to make any direct comparison on long-term procedure survival and clinical outcomes between BMS and fixation. The authors consider LDFF the first-line surgical treatment in case of a symptomatic fragmentous OLT amendable for fixation which is not responding to conservative therapy. This is case as other surgical treatment options remain available in case of treatment failure [20]. According to the senior author, the reason for failure of the 3 revised ankles (in two patients) is partial non-union of the osteochondral fragment. This raises a clinical question what baseline patient and lesion factors may influence union and warrants further investigation.

Fixation for OLTs can only be considered in specific cases to be amendable for fixation, with a fragment of sufficient dimensions and good cartilage coverage [19]. It should be stated that arthroscopic fixation of OLTs can be technically demanding and that it to be reserved for experienced arthroscopists. In addition to the previously described LDFF technique, the authors note a supplemental technique recommendation [8]. To gain sufficient access to the lesion and to achieve an adequate perpendicular compression force on the fragment, a third portal may be necessary for fixation. A portal 1–2 cm superiorly from to the standard working portals, depending on the lesion location, can be made for this purpose. Combined with the ankle in full plantar flexion (for anterior and central lesions) or dorsiflexion (for posterior lesions), optimal access and a perpendicular screw insertion angle can be achieved, which is important for fragment union and limiting the chance for osteolytic changes without the need for an additional osteotomy [13].

To the knowledge of the authors, this is the first study reporting long-term clinical outcomes following arthroscopic fixation of OLTs. All patients were treated at a single center which is accredited as an (inter)national expert center for the diagnosis and treatment of ankle cartilage injuries. Patients were prospectively followed and data were collected by independent investigators not involved in patient care to limit observer bias. All radiological measurements were conducted by two independent raters with excellent inter-rater reliability.

The present study is not without its limitations. First, 20% of eligible ankles were lost to follow-up. Lost to follow-up is a known problem in long-term follow-up studies [7]. It could be interpreted as a good sign, that patient need no further care for their ankle, nevertheless they might as well have looked for care elsewhere. Second, as previously described by Lambers et al. [10], it should be noted that clinical outcome measures were not available for all patients at every follow-up moment due to a historical change in the outcome measures used for the clinical assessment. Third, the present study did not include a long-term radiological follow-up to assess the presence of degenerative changes in the tibiotalar joint. Fourth, the present study included a limited number of patients, limiting the statistical power.

The present study addresses the paucity of literature concerning the long-term clinical outcomes as well as the clinical sustainability of the procedure. Fixation of fragmentous OLTs should always be considered by the treating physician and should be made in the context of an individualized treatment algorithm which includes patient and lesion characteristics [20]. This study underlines the need for prospective research assessing clinical outcomes of fixation techniques, both open and arthroscopically, with sufficient statistical power to provide further evidence for its clinical efficacy in OLT treatment as well as to assess prognostic factors associated with treatment success.

## Conclusion

Arthroscopic LDFF for fixable chronic primary OLTs results in a long-term procedure survival rate of 87%. Clinically, excellent and sustained pain reduction and patient-reported outcomes were observed. These results indicate that surgeons may consider arthroscopic fixation for a fragmentous OLT.

## Appendix 1 (Table 3)

**Table 3:** Baseline demographics and treatment characteristics for failure cases

	Patient characteristics					Lesion characteristics			Treatment characteristics			
Case	Sex	Age at surgery, years	BMI	Laterality	Injury etiology	Lesion location	Lesion size, mm	Morphology	Screw-type	Reason Revision Surgery	Revision Surgery (procedure)	Revision Surgery (FU time)
1	F	16	19.4	L	Traumatic	Zone 1	AP: 11 ML: 9 Depth: 5	Fragment, w/sub-fragmentary cyst	Bio-absorbable	Symptomatic OLT, partial non-union fragment	Arthroscopic debridement and BMS	14 months
2	M	16	19	L	Traumatic	Zone 3	AP: 19 ML: 11 Depth: 10	Fragment w/sub-fragmentary cyst	Bio-absorbable	Symptomatic OLT, non-union fragment	Open LDFF	20 months
				R	Traumatic	Zone 3	AP: 25 ML: 12 Depth: 10	Crater w/sub-fragmentary cysts	Chondro-darts	Symptomatic OLT, non-union fragment	Open LDFF	36 months

*FU* follow-up, *F* female, *M* male, *L* left, *R* right, *AP* anterior–posterior, *ML* medial–lateral
